# Administration of High-Dose Methylprednisolone Worsens Bone Loss after Acute Spinal Cord Injury in Rats

**DOI:** 10.1089/neur.2021.0035

**Published:** 2021-12-08

**Authors:** Yuanzhen Peng, Wei Zhao, Yizhong Hu, X. Edward Guo, Jun Wang, Ke Hao, Zhiming He, Carlos Toro, William A. Bauman, Weiping Qin

**Affiliations:** ^1^ National Center for the Medical Consequences of Spinal Cord Injury, James J. Peters VA Medical Center, Bronx, New York, USA.; ^2^ Departments of Medicine, Icahn School of Medicine at Mount Sinai, New York, New York, USA.; ^3^ Rehabilitation and Human Performance, Icahn School of Medicine at Mount Sinai, New York, New York, USA.; ^4^ Department of Biomedical Engineering, Columbia University, New York, New York, USA.; ^5^ Department of Neurology, Icahn School of Medicine at Mount Sinai, New York, New York, USA.; ^6^ Department of Genetics and Genomic Sciences, Icahn School of Medicine at Mount Sinai, New York, New York, USA.; ^7^ College of Dentistry, New York University, New York, New York, USA.

**Keywords:** bone loss, bone resorption, methylprednisolone, receptor activator of NF-κB ligand, spinal cord injury

## Abstract

The administration of high-dose methylprednisolone (MP) for 24–48 h after traumatic spinal cord injury (SCI) has been shown to improve functional recovery. The known adverse effects of MP on skeletal muscle and the immune system, though, have raised clinically relevant safety concerns. However, the effect of MP administration on SCI-induced bone loss has not been evaluated to date. This study examined the adverse effects of high-dose MP administration on skeletal bone after acute SCI in rodents. Male rats underwent spinal cord transection at T3–T4, which was followed by an intravenous injection of MP and subsequent infusion of MP for 24 h. At 2 days, animals were euthanized and hindlimb bone samples were collected. MP significantly reduced bone mineral density (−6.7%) and induced deterioration of bone microstructure (trabecular bone volume/tissue volume, −18.4%; trabecular number, −19.4%) in the distal femur of SCI rats. MP significantly increased expression in the hindlimb bones of osteoclastic genes receptor activator of nuclear factor-κB ligand (RANKL; +402%), triiodothyronine receptor auxiliary protein (+32%), calcitonin receptor (+41%), and reduced osteoprotegerin/RANKL ratio (−72%) compared to those of SCI-vehicle animals. Collectively, 1 day of high-dose MP at a dose comparable to the dosing regimen prescribed to patients who qualify to receive this treatment approach with acute SCI increased loss of bone mass and integrity below the level of lesion than that of animals that had SCI alone, and was associated with further elevation in the expression of genes involved in pathways associated with osteoclastic bone resorption than that observed in SCI animals.

## Introduction

Patients with spinal cord injury (SCI) often experience devastating neurological impairments, and they frequently require complex long-term multi-disciplinary care, which causes substantial individual and societal burden and socioeconomic impact.^1^ Glucocorticoid methylprednisolone (MP) is a U.S. Food and Drug Administration–approved agent that is being used in appropriate clinical settings in an attempt to improve function after acute SCI, in part because of its ability to inhibit lipid peroxidation and inflammation.^2–5^ The results of the Second National Acute Spinal Cord Injury Study (NASCIS) II and III clinical trials demonstrated that when begun within 8 h after SCI in patients, administration for 24–48 h of high-dose MP has been demonstrated to improve functional neurological recovery.^4–6^ However, a number of adverse effects of this treatment in patients with acute SCI have been reported, including immunosuppression, susceptibility to infection, wound complications, gastric bleeding, sepsis, diabetic complications, pneumonia, and acute corticosteroid myopathy.^4,7–10^

Recent animal work from our group indicated that the administration of high-dose MP for 24 h reduced muscle size and increased atrophy-related gene expression in spinal-cord–injured rats.^11,12^ Glucocorticoid-induced osteoporosis and the associated increase in risk of fracture are common, severe adverse effects related to chronic glucocorticoid treatment.^13^ It remains unclear whether short-term administration of high-dose MP results in a greater degree of bone loss after acute SCI than does SCI alone. Although specific adverse effects of MP-induced bone loss have not been studied in patients with SCI, one can envision reduced ability to transfer, operate a wheelchair, and benefit from using new technologies for ambulation (e.g., exoskeletal-assisted ambulation) because of steroid-induced muscle loss above the level of neurological lesion and worsened osteoporosis that will predispose to an increased risk of lower-extremity fracture. Support for these concerns arise from findings in SCI rats in which high-dose MP markedly reduced the muscle weights of neurologically intact triceps and the paralyzed gastrocnemius, soleus, and plantaris muscles when compared with the respective muscles obtained from an SCI vehicle group.^11^

Bone loss arises largely through the net loss of bone by accelerated breakdown by osteoclasts and reduced formation of bone substance by osteoblasts.^14,15^ Bone resorption is stimulated by cells of the osteoblast lineage by the release of receptor activator of nuclear factor-κB ligand (RANKL), which stimulates differentiation and activity of osteoclasts.^14^ Sclerostin, a product of the sclerostin (*SOST*) gene, is mainly produced by osteocytes and is a potent inhibitor of bone formation by downregulation of the Wnt signaling pathway in bone.^15–17^ In the initial phase of glucocorticoid-induced bone loss, upregulation of RANKL has been suggested to explain the rapidly increased rates of bone resorption observed.^18,19^ Whether a relatively brief period of high-dose MP administration prescribed at the time of traumatic SCI in appropriate patients in an attempt to improve functional outcome results in significant changes in the expression of osteoclastic resorption genes (e.g., RANKL, triiodothyronine receptor auxiliary protein [TRAP], intergrin, and calcitonin receptor [CTR]) has not been reported.

SCI causes mechanical unloading of skeletal regions immobilized by paralysis and extensive loss of muscle mass and sublesional bone.^20–23^ Marked early bone loss is detected mainly at the distal femur and proximal tibia, where fracture predominantly occurs.^24–26^ As a consequence of acute SCI, abnormal skeletal unloading dysregulates bone metabolism with a marked depression of osteoblastic bone formation as well as a profound increase in osteoclastic bone resorption.^20,21,27–29^ More recently, we demonstrated that bone loss and deterioration of trabecular bone microstructure occur as early as 2 days after neurologically motor-complete SCI; such bone defects are likely the result of higher levels of osteoclastic resorption, mainly driven by the marked increase in RANKL gene expression.^30^

The purpose of this study was to evaluate the adverse effects of administration of MP on SCI-induced bone loss and examine the molecular mechanism involved. We hypothesize that administration of high-dose MP for 24 h further increases the rapid bone loss caused by acute SCI. In the present study, the effects of MP administered in the doses and temporal sequence prescribed in an effort to improve neurological function after acute SCI on bone mass and microstructure were characterized. Next, the changes of gene expressions strongly linked to bone metabolism were examined. A rat model of complete spinal cord transection at the midthoracic level was used for these studies. Because ∼80% of persons with SCI are male, male rats were used in these studies.^31^

## Methods

### Animals, surgery, drug administration, and tissue collection

All animals were maintained on a 12:12-h light/dark cycle with lights on at 7:00 am in a temperature-controlled (20°C ± 2°C) vivarium, and all procedures were approved by the Institutional Animal Care and Use Committee, James J. Peters Veteran Affairs Medical Center (New York, NY).

#### Choice of the animal model of spinal cord injury

In NASCIS II and III clinical trials, MP shows significant benefit on motor function recovery in persons with both neurologically complete (e.g., plegic) and incomplete (e.g., paretic) SCI, although overall neurological recovery was considerably greater in patients with incomplete versus complete injuries.^4,5,8^ Although a contusion-injured spinal cord model is more representative of SCI clinical presentations, the complete SCI model was chosen in this study because it allows us to: 1) create a consistent and defined initial injury that minimizes variation from animal to animal and 2) clearly distinguish the initial injury (caused by a direct cut) from subsequent secondary injury. Our group has performed considerable work in the study of musculoskeletal disorders after SCI using complete SCI animal models.^11,12,17,30,32–36^

As an example of our past work, a rat model of SCI induced by complete spinal cord transection has been successfully used by our group to test adverse effects of MP administration on skeletal muscle^11,12^ and bone.^30^ We found that 1 day of MP at a dose comparable to those routinely used in clinical practice immediately after SCI resulted in marked atrophy of functionally intact muscle above the level of lesion, worsened atrophy of paralyzed muscle,^11,12^ and increased loss of bone mass and integrity^30^ below the level of lesion over and above that induced by SCI alone. Thus, a complete SCI model induced by spinal cord transection has provided the investigators a practical, consistent, and reliable platform to address the side effects that result from MP or other interventions on musculoskeletal biology.

Spinal cord transection surgery was performed as previously described.^11,12,17,30,32–36^ In brief, 9-week-old Wistar rats (Charles River, Wilmington, MA) were anesthetized by inhalation of isofluorane (3–5%),, and hair was removed with a clipper. Skin over the back was cleaned with betadine and isopropyl alcohol. After making a midline incision, the spinal cord at the site of transection (T3–T4) was visualized by laminectomy, and the spinal cord was transected with microscissors. The space between transected ends of the spinal cord was filled with surgical sponge, and the wound was closed in two layers with suture. Urine was manually expressed three times daily until automaticity developed, then urine was expressed as needed. Baytril was administered for the first 3–5 days post-operatively, then administered as needed for a sign of cloudy or bloody urine or wound infection. Sham-transected animals received only a laminectomy.

Immediately after spinal cord transection, animals were administered an intravenous injection of freshly prepared MP (30 mg/kg; Pfizer Inc., New York, NY) or vehicle (propylene glycol) by tail vein injection, followed by implantation of an Alzet 2001 pump (Durect Co, Cupertino, CA), which provided a 24-h infusion of MP at 5.4 mg/kg/h (SCI-MP), or vehicle (propylene glycol; SCI) into the subcutaneous space. The dosing of MP used in this protocol corresponds on a milligram per kilogram basis to that prescribed by the Bracken protocol.^6,11^ Sham-transected (sham) animals underwent laminectomy and implantation of the same Alzet pumps, with vehicle (propylene glycol) infusion; the other control group had a spinal cord transection, as described above, and infusion with vehicle (SCI-vehicle). Numbers per group were: sham-SCI, *n* = 10; SCI, *n* = 10; SCI-MP, *n* = 12. Animals from the sham-SCI and SCI-vehicle groups have been included in a previous report.^30^

Body weights were recorded before spinal cord transection (pre-operative body weight) and daily after SCI. Body weight at euthanization was normalized relative to pre-operative body weight. Two days after SCI, animals were euthanized by inhalation of isofluorane before harvesting of tissue for study. The leg was removed using a sterile technique; careful dissection was performed to free the head of the femur from the pelvis. To preserve bone for micro computed tomography (μCT; *n* = 6–7 per group), the left leg was removed and placed into tubes containing 4% paraformaldehyde (PFA) overnight, after which the PFA was replaced with 70% ethanol for storage. The right femur and tibia (*n* = 4–5 per group) were placed in ice-cold minimum essential alpha medium and then immediately processed for extraction of total RNA from whole bone, as described below.

### Dual-energy X-ray absorptiometry

Areal BMD measurements were performed on excised hindlimbs (*n* = 10–12 animals per group) by using a small animal dual-energy X-ray absorptiometer (DXA; Lunar PIXImus; GE Medical Systems, Madison, WI), as previously described.^17,32–35,37,38^ Hindlimbs were positioned on the DXA platform with the knee flexed at an angle of 135 degrees, and DXA images were acquired with Lunar Pixmus software. Before analysis of samples, the DXA machine was calibrated using a phantom according to the manufacturer's recommendation. The metaphysis of the distal femur and proximal tibia were selected as regions of interest (ROIs). The coefficient of variation for repeated measurements for the ROI was ∼1.5%.

### Micro computed tomography analysis of bone microarchitecture

Bone architecture of the distal femur was assessed by a Scanco μCT scanner (vivaCT 80; Scanco Medical AG, Wangen-Brüttisellen, Switzerland) at 21 mm isotropic voxel size, as previously described^17,32,33,35,39,40^ (also see the Supplementary Materials and Methods for greater details). Image reconstruction and three-dimensional (3D) quantitative analysis were performed using software provided by Scanco. Scans were initiated at the distal end of the femur and extended to the center of the femur for a total of ∼777 slices (∼16.3 mm). Trabecular ROIs consisted of 189 slices (∼3.969 mm), beginning 0.5 mm proximal to the growth plate and continuing in a proximal direction, were included in the bone analysis. Cortical ROIs consisting of 100 slices were located at the center of the femur (∼2.1 mm) and analyzed. Standard nomenclature and methods for bone morphometric analysis were used.^17,39^

Mechanical properties at the distal femur trabecular bones were estimated from micro finite element analysis (μFEA), following the manufacturer's recommended procedures, as previously described.^21,35,41–43^ Briefly, μFEA models were produced by converting each bone voxel to an eight-node brick element. Bone tissue was subjected to applied uniaxial compression, with an elastic modulus of 15 GPa and Poisson's ratio of 0.3 for each element. A linear elastic analysis was used to estimate bone stiffness.

### Extraction of total RNA from bone

Total bone RNA was extracted, as previously described, with some modifications.^30,44^ Briefly, long bones were dissected free of soft tissues, and bone marrow was flushed away with phosphate-buffered saline using a 27G½ needle syringe. Bone samples (∼1 g) were longitudinally cut into small pieces and then digested three times with 2 mg/mL of collagenase type I (>150 U; 20 mL; Gibco, Amarillo, TX), one time with 5 mM of ethylenediaminetetraacetic acid (EDTA; 10 mL; Sigma-Aldrich, St. Louis, MO), and one more time with the collagenase and EDTA, each for 25 min on a shaker with rotation at 150 rpm at 37^0^C. After the digestions, bone samples were crushed using a mortar and pestle in liquid nitrogen. RNA was extracted from the lysate using the TRizol reagent (Sigma-Aldrich), according to the manufacturer's instructions.

### Quantitative polymerase chain reaction

Real-time polymerase chain reaction (PCR) was used for the determination of messenger RNA (mRNA) levels, as described previously.^17,34,37^ First, 1 μg of total RNA was used to synthesize first-strand complementary DNA (cDNA) by the High Capacity cDNA Reverse Transcription Kit (Applied Biosystems [ABIO], Foster City, CA). Quantitative PCR (qPCR) was performed with an ABI Via 7 thermal cycler using ABI Taqman 2X PCR mix and ABI Assay on Demand qPCR primers. Changes in expression were calculated using the 2^-ΔΔCt^ method using 18S RNA as the internal control.^17,34,37^

### Statistical analysis

Standard power analyses were used to determine the requisite minimum number of animals to ensure sufficient statistical power, as described.^45^ Data are expressed as mean ± standard deviation (SD). The number of independent samples (*n*) is provided in the legend of each figure. The statistical significance of differences among means was tested using one-way analysis of variance and a Newman-Keuls *post hoc* test to determine the significance of differences between individual pairs of means using a *p* value of 0.05 as the cutoff for significance. Statistical calculations were performed using Prism software (version 4.0c; GraphPad Software, La Jolla, CA).

## Results

### Body weights, bone mass, and microstructure

**FIG. 1.**
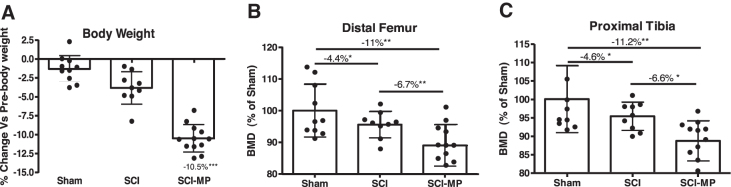
Effects of MP on bone mass after acute SCI. (**A**) Changes in body mass are shown. Body weights at euthanizing were normalized relative to body weight before spinal cord transection (pre-operative body weight). (**B,C**) Areal bone mineral density (BMD) measurements in each group are shown at the distal femur (B) and proximal tibia (C). Data are expressed as mean ± SD. Sham group, *n* = 10; SCI group, *n* = 9, SCI-MP group, *n* = 12. Significance of differences was determined using one-way analysis of variance with a Newman-Keuls test *post hoc*. *p < 0.05; **p < 0.01; ***p < 0.001 versus the indicated group; NS indicates no significant difference. MP, methylprednisolone; SCI, spinal cord injury; SD, standard deviation.

Body weights were significantly lower (−10.5%, *p* < 0.01) in the SCI-MP group at 2 days, compared to the SCI-vehicle group (Fig. 1A).

In a parallel study, we demonstrated that motor-complete SCI causes rapid loss of bone mass and deterioration of trabecular bone microstructure as early as 2 days after injury.^30^ In the present study, we asked whether systemic administration of MP could induce further bone loss in rats that received a complete spinal cord transection. Using a small animal DXA, bone mineral density (BMD) was determined. At the distal femur (Fig. 1B), SCI resulted in a loss of BMD by −4.4% (*p* < 0.05) as compared to SCI-vehicle, and SCI-MP rats showed an additional loss of BMD by −6.7% (*p* < 0.01) as compared to the SCI-vehicle group, resulting in a total of −11% reduction in BMD (*p* < 0.01) compared to sham control animals. An almost identical BMD pattern was detected at the proximal tibia (Fig. 1C), in which SCI resulted in a loss of BMD by −4.6% (*p* < 0.05) as compared to SCI-vehicle, and SCI-MP rats had an additional loss of BMD by −6.6% (*p* < 0.05) as compared to the SCI-vehicle group, resulting in a total of −11.2% lower BMD (*p* < 0.01) in SCI-MP rats compared to sham control animals.

**FIG. 2.**
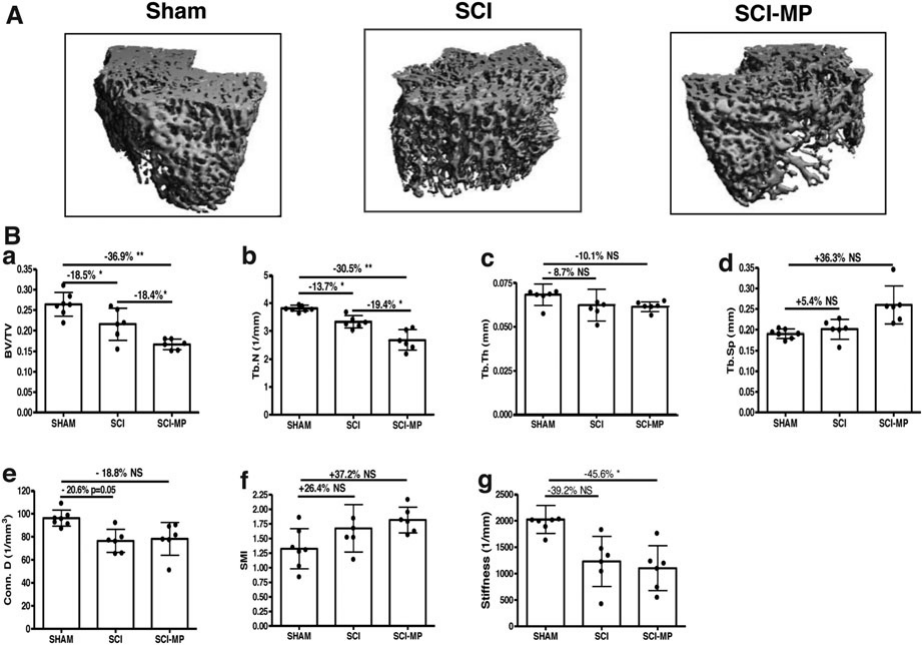
Effects of MP on trabecular microstructure after acute SCI. (**A**) Representative μCT 3D images of trabecular microarchitecture are displayed. (**B**) Measurements are shown for: (a) trabecular bone volume per total tissue volume (BV/TV), (b) trabecular number (Tb.N; mm^−1^), (c) trabecular separation (Tb.Sp; mm), (d) connectivity density (conn.D; mm^−3^), and (e) structure model index (SMI). (f) Bone stiffness was estimated from micro finite element analysis (μFEA). Data are expressed as mean ± SD. Sham group, *n* = 6; SCI group, *n* = 6, SCI-MP group, *n* = 7. Significance of differences was determined by using one-way analysis of variance with a Newman-Keuls test *post hoc*. *p < 0.05; **p < 0.01 versus the indicated group; NS indicates not significant. 3D, three-dimensional; μCT, micro computed tomography; MP, methylprednisolone; SCI, spinal cord injury; SD, standard deviation.

Bone architecture was examined by high-resolution μCT to assess changes in trabecular bone of the distal femur (Fig. 2). At 2 days post-injury, SCI-MP greatly reduced trabecular bone volume (bone volume/tissue volume [BV/TV], −36.9%, *p* < 0.01; Fig. 2B-a), trabecular bone number (Tb.N; −30.5%, *p* < 0.01; Fig. 2B-b), trabecular thickness (Tb.Th; −10.1%; Fig. 2B-c), connectivity density (Conn.D; −18.8%, Fig. 2B-e), and bone stiffness (−45.6%, *p* < 0.05; Fig. 2B-g), whereas there were increasing trabecular separation (Tb.Sp; +36.3%; Fig. 2B-d) and structure model index (SMI; +37.2%; Fig. 2B-f) compared to sham control. Importantly, SCI-MP rats showed −18.4% and −19.4% reductions in BV/TV (*p* < 0.05) and Tb.N (*p* < 0.05) compared to the SCI-vehicle group, respectively. The magnitude of MP-induced changes on other parameters of bone architecture was greater than that of the SCI-vehicle group, although these changes did not reach statistical significance.

**FIG. 3.**
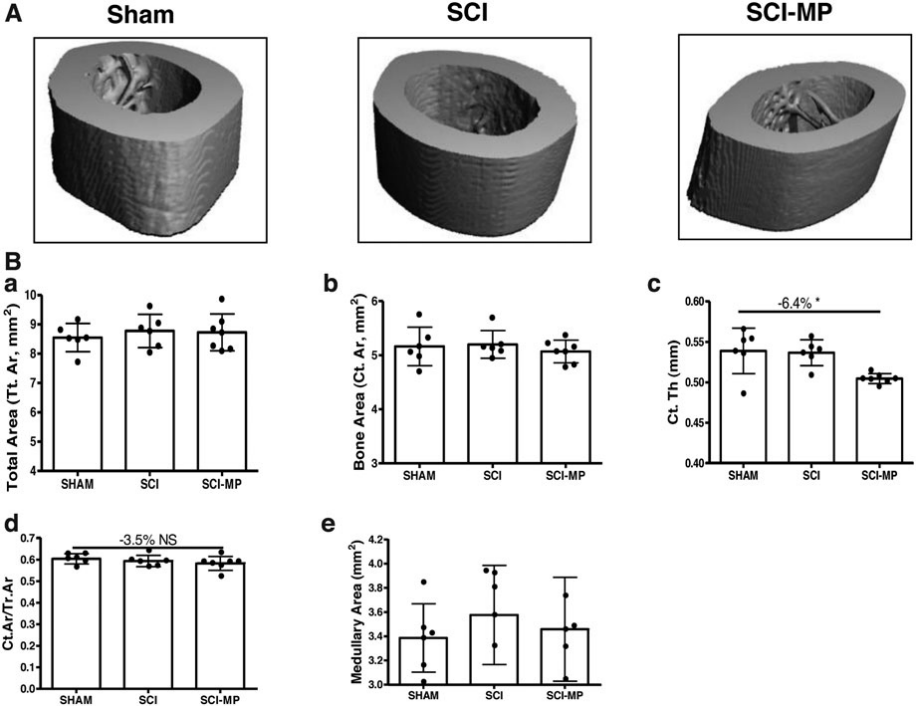
Effects of MP on cortical architecture of the femur midshaft after acute SCI. (**A**) Representative μCT 3D images of cortical microarchitecture are displayed. (**B**) Cortical bone volume over total tissue volume (BV/TV). Data are expressed as mean ± SD. Sham group, *n* = 6; SCI group, *n* = 6, SCI-MP group, *n* = 7. Significance of differences was determined by using one-way analysis of variance with a Newman-Keuls test *post hoc*. *p < 0.05 versus the indicated group. 3D, three-dimensional; μCT, micro computed tomography; MP, methylprednisolone; SCI, spinal cord injury; SD, standard deviation.

Cortical bone structure at the femur midshaft was also examined by high-resolution μCT (Fig. 3). There was no significant change in structural index from SCI animals administered MP or vehicle, compared to those from the sham group.

### Bone gene expression

**FIG. 4.**
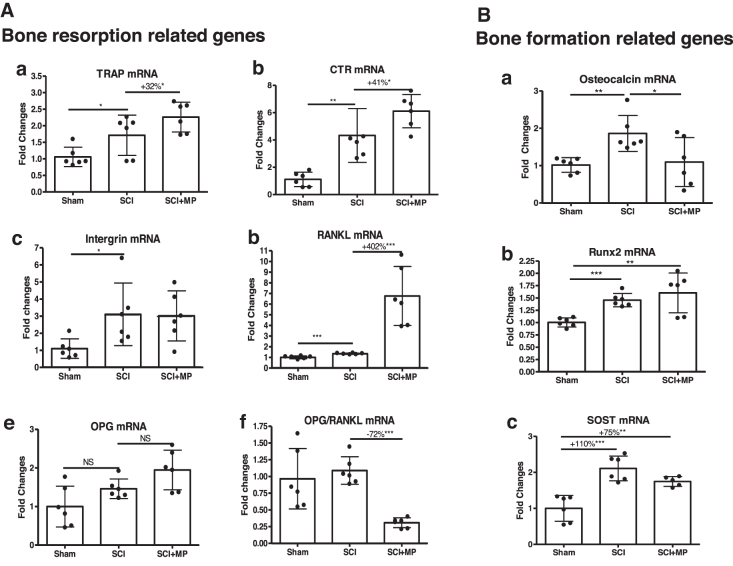
Effects of MP on bone gene expressions after SCI. Using RNA extracted from long bones, gene expressions related to bone resorption (**A**) and formation (**B**) were determined by real-time PCR analysis. (A-a) TRAP, (A-b) CTR, (A-c) intergrin β3, (A-d) RANKL, (A-e) OPG, (A-f) OPG/RANKL ratio, (B-a) osteocalcin, (B-b) Runx2, and (B-c) SOST. Gene expression was normalized by 18s. Data are expressed as mean ± SD. Sham group, *n* = 6; SCI group, *n* = 6; SCI-MP group, *n* = 6. Significance of differences was determined by using one-way analysis of variance with a Newman-Keuls test *post hoc*. *p < 0.05; **p < 0.01; ***p < 0.001 versus the indicated group; NS indicates no significant difference. CTR, calcitonin receptor; MP, methylprednisolone; OPG, osteoprotegerin; PCR, polymerase chain reaction; RANKL, receptor activator of nuclear factor-κB ligand; Runx2, Runt-related transcription factor 2; SCI, spinal cord injury; SD, standard deviation; SOST, sclerostin; TRAP, triiodothyronine receptor auxiliary protein.

To understand molecular mechanisms underlying MP-induced bone loss after acute SCI, total RNA from whole bone of hindlimb was extracted and gene expression responsible for bone resorption and formation were analyzed by qPCR analysis. MP significantly increased osteoclastic markers TRAP (+32%, *p* < 0.05; Fig. 4A-a) and CTR (+41%, *p* < 0.05; Fig. 4A-b) as compared to those in SCI-vehicle animals. Notably, MP led to a marked higher level of expression for RANKL (+402%, *p* < 0.001; Fig. 4A-d) and a lower ratio of osteoprotegerin (OPG)/RANKL (−72%, *p* < 0.001; Fig. 4A-f), as compared to those in SCI-vehicle animals. Although mRNA expression of Runt-related transcription factor 2 (Runx2) and SOST (two genes related to bone formation) remained high in SCI-MP animals (Fig. 4B-b,c), osteocalcin expression was significantly reduced in SCI-MP animals compared to that in SCI-vehicle animals (*p* < 0.05; Fig. 4B-a).

## Discussion

We recently reported that sublesional loss of BMD in rodents is as great as 4.4–4.6% within the first 2 days of acute SCI,^30^ a degree of bone loss that has been observed after 1 year of menopause in women who are not taking osteoprotective drugs.^46^ It is well characterized that patients who were treated chronically with glucocorticoids have several toxic effects, including an appreciable bone loss with an associated increased risk of fragile fracture.^18^ The glucocorticoid-mediated fractures occur at higher BMD values than occur in post-menopausal osteoporosis.^47^ The findings from the present study demonstrated, for the first time, that administration for 1 day of MP at doses equivalent to those clinically prescribed to increase function after acute SCI rapidly caused further adverse side effects on the sublesional skeleton, leading to increased loss of bone mass and decreased structural integrity over and above that which results from spinal cord transection alone. Thus, skeletal deterioration associated with the administration of high-dose MP in patients with acute SCI is clearly a new and important concern that should be appreciated and taken into consideration in future clinical management in the attempt of clinicians to improve functional recovery.

The detrimental effect of high-dose glucocorticoid administration on bone results from direct effects of this class of agents on osteoblasts, osteoclasts, and osteocytes. Elevated glucocorticoid levels stimulate the synthesis of the receptor activator of nuclear factor-κB (RANK) and RANKL by pre-osteoblast/stromal cells, supporting osteoclast differentiation, viability, activity with resultant net bone resorption.^48^ Glucocorticoids also suppress the production of osteoprotegerin, an inhibitor of osteoclast differentiation from marrow hematopoietic cells of the macrophage lineage. With chronic use, the predominant effect of glucocorticoids on the skeleton is suppression of bone formation mediated by direct inhibition of osteoblast proliferation and differentiation and by an increase in the apoptosis rates of mature osteoblasts and osteocytes.^49–52^

In the current study, MP administration was associated with early increase in expression of several genes linked to bone catabolism, such as TRAP and RANKL, as well as a lower ratio of OPG/RANKL. Of note, MP robustly enhanced the expression of RANKL by 4-fold, which represents a key mechanism through which MP enhances osteoclast function and promotes bone resorption in the setting of acute SCI. Such a prompt and marked increase in levels of RANKL expression contributes to the rapid deterioration of bone mass and trabecular microstructure that was evident in SCI rats only 2 days after MP administration. The absence of changes in expression of Runx2 and SOST suggest that bone formation and osteocyte function by this mechanism are not compromised with administration of MP in acute SCI, but the observed depression in expression in osteocalcin, a marker of bone formation, likely plays a role in increased net bone resorption. The long-term effects of MP use in bone formation and osteocyte function after SCI remains to be determined.

This study has a number of strengths, including a validated rodent model of acute SCI in which the additive effects of high-dose glucocorticoid administration could be demonstrated on bone below the level of lesion, and we have identified a likely potential mechanism responsible for MP-induced osteoclastic bone resorption. However, this work has limitations. Only male rats and at one time point (2 days post-injury) were studied. From our past work, we found a lasting effect of a significant reduction in sublesional muscle weight (e.g., that of soleus) in the SCI-MP group 14 days after MP administration compared to soleus muscle weight in the group that had only SCI.^11^ It thus remained of interest and relevance to investigate whether a brief period of MP administration at the time of acute SCI to improve function would result in similar deleterious changes to the sublesional skeleton and bone metabolism both in male and female animals. In the work herein, young adult rats with skeletons that were still maturing and accruing bone mass were studied. As such, it would also be worthwhile to confirm our findings in older animals that are skeletally mature. Additionally, the present study demonstrated the interesting finding that MP accelerates the bone loss observed after SCI, and future investigations can address another interesting question as to how SCI plays a role in MP-induced osteopenia; for this purpose, the study can include a non-SCI group of animals that receive only MP administration, which would permit one to quantitate the degree of bone loss that MP administration causes alone compared to the additive effect of MP and SCI on bone loss, as well as define the deleterious skeletal cellular and molecular changes that are distinctive for the combination of MP and SCI versus MP alone. Last, an animal model with neurologically motor-complete SCI was used in the present study. In future work, we will adopt a more clinically relevant rat model with incomplete contusion SCI to further evaluate effect of MP or other interventions with the potential to mitigate the adverse side effects associated with MP administration on the wound-healing response (e.g., lesion/cavity volume and injury-related cellular reactivity) and functional outcomes after SCI.

Collectively, our findings reported herein indicate that the administration of high-dose MP for 24 h resulted in the acute effect to induce further deterioration of bone mass and integrity in hindlimb bones of SCI rats than that of SCI alone. It appears that one mechanism for our observation is through the upregulation of osteoclastic resorption-related gene expression. The study provides novel experimental evidence in support of the deleterious effects of MP on the sublesional skeleton when administered after acute SCI, in addition to other clinical concerns associated with high-dose MP use. With this in mind, one of the ongoing efforts in our laboratory is to develop a novel nanotechnology-based targeting delivery system that can specifically deliver MP to the spinal cord lesion, but not to other parts of the body, which would serve to maximize the desired neuroprotection while minimizing adverse systemic side effects in other organs, including those of the musculoskeletal system.

## Data Archiving

The data sets generated and/or analyzed during the current study are available from the corresponding author on reasonable request.

## Statement of Ethics

We certify that all applicable institutional and governmental regulations concerning the ethical use of animals were followed during the course of this research.

## Supplementary Material

Supplemental data

## Abbreviations Used

3D: three-dimensional
BMD: bone mineral density
BV/TV: bone volume/tissue volume
cDNA: complementary DNA
Conn.D: connectivity density
μCT: micro computed tomography
CTR: calcitonin receptor
DXA: dual-energy X-ray absorptiometer
EDTA: ethylenediaminetetraacetic acid
μFEA: micro finite element analysis
MP: methylprednisolone
mRNA: messenger RNA
OPG: osteoprotegerin
PCR: polymerase chain reaction
PFA: paraformaldehyde
qPCR: quantitative PCR
RANK: receptor activator of nuclear factor-κB
RANKL: receptor activator of nuclear factor-κB ligand
ROIs: regions of interest
Runx2: Runt-related transcription factor 2
SCI: spinal cord injury
SD: standard deviation
SMI: structure model index
SOST: sclerostin
Tb.N: trabecular bone number
Tb.Sp: trabecular separation
Tb.Th: trabecular thickness
TRAP: triiodothyronine receptor auxiliary protein
