# Supplementary material for: Administration of High-Dose Methylprednisolone Worsens Bone Loss after Acute Spinal Cord Injury in Rats
Source: Neurotrauma Rep. 2021 Dec 8;2(1):592–602. doi: 10.1089/neur.2021.0035 (PMC8742306; doi:10.1089/neur.2021.0035)
Supplement: Supplemental data [file Supp_DataS1.docx]

**Supplemental Materials and Methods**

***1.*** *MicroCT Analysis of Trabecular Architecture*

To evaluate trabecular architecture of the distal femur, microCT was performed on fixed bones, as described previously using a Scanco μCT scanner and a 21-μm voxel size (26, 32, 40, 41). The femur was placed in a holder and scanned non-destructively by using a Scanco µCT scanner (vivaCT 80; Scanco Medical AG, Bassersdorf, Switzerland) at 21 µm isotropic voxel size (the highest resolution) with X-ray source power of 55 kV and 145 µA and integration time of 300 milliseconds. The trabecular microstructure of the distal femur was evaluated. The scanned grey-scale images were processed by using a low-pass Gaussian filter (sigma = 0.8, support = 1) to remove noise, and a fixed threshold of 220 was used to extract the mineralized bone from soft tissue and marrow phase. The reconstruction and 3D quantitative analyses were performed by using software provided by Scanco. The same settings for scan and analysis were used for all samples.

Scans were initiated at the distal end of the femur and extended to the center of the femur, for a total of approximately 777 slices (~16.3 mm). Trabecular regions of interest consisted of 189 slices (~3.969 mm), beginning 0.5 mm proximal to the growth plate and continuing in a proximal direction, were included in the bone analysis. Cortical regions of interest consisted of 100 slices at the proximal end of the scans, located ~2.1 mm at the center of the femur, were also analyzed. Cancellous bone was separated from the cortical regions by semi-automatically drawn contours. The following 3D indices in the defined ROI were analyzed: bone volume (BV, mm^3^), tissue (cortical and marrow) volume (TV, mm^3^), relative bone volume over total volume (BV/TV, %), trabecular number (Tb.N, mm^-1^), trabecular thickness (Tb.Th, µm), trabecular separation (Tb.Sp, µm), connectivity density (Conn.Dn, mm^-3^), structure model index (SMI, ranges from 0 to 3 with 0 = platelike and 3 = rodlike).

*2. Quantitative PCR.*

The following osteoblastic and osteoclastic primers are purchased from ABI TaqMan Gene Expression Assay: RANKL (Rn00589289_m1),TRAP (Rn00569608_m1), Intergrin β3 (Rn01763790_m1), calcitonin receptor (CTR) (Rn00587525_m1), Runx2 (Rn01512296_m1)*,* osteocalcin (Rn00566386_g1), BSP (Rn00561414_m1),. SOST primer is a customer-made one: SOST-R: ATCTTTGGCGTCATAGGGATGGTG; SOST-F:CTTCAGGAATGATGCCACAGAGGT.
